# Changes in HDAC Expression and Activity by Oolongtheanin Digallate and Theasinensins and the Onset of Apoptosis

**DOI:** 10.3390/molecules31122101

**Published:** 2026-06-15

**Authors:** Johannes Gröne, Julian Alfke, Marco Fortmann, Uta Kampermann, Mustafa Qutaiba Ibrahim Masoodi, Hans-Ulrich Humpf, Melanie Esselen

**Affiliations:** Institute of Food Chemistry, University of Münster, Corrensstr. 45, 48149 Münster, Germany

**Keywords:** polyphenols, epigallocatechin gallate, apoptosis, histone deacetylases, proteomics, bioactivity

## Abstract

Epigallocatechin gallate (EGCG) is the major polyphenol in green tea and is frequently used in food supplements. In recent years, numerous studies have highlighted the bioactivity of polyphenols beyond their established role as radical scavengers. However, EGCG is highly unstable in slightly basic solutions such as cell culture medium. It therefore remains unclear whether the biological effects attributed to EGCG are caused by the parent compound itself or by its oxidation products, including the dimers examined here. In this study, the effects of EGCG focusing on apoptosis induction and histone deacetylases (HDAC) were compared with those of its major oxidation products, theasinensin A (TSA), theasinensin D (TSD), and oolongtheanin digallate (OTDG), in the human hepatocellular carcinoma cell line HepG2. The induction of cellular pathways involved in apoptosis was investigated using several in vitro biochemical approaches. Transcriptional analysis of apoptosis-associated genes revealed distinct expression profiles, and caspase activities were differentially affected by the test compounds. HDAC activity in nuclear protein extracts was significantly reduced after incubation with the stabilized oxidation products, whereas no comparable HDAC-inhibitory effect was observed after direct incubation of HepG2 cells. Nevertheless, HDAC gene expression, particularly of class I isoforms, was modulated by the test compounds in the low micromolar range. These effects diminished at concentrations associated with the onset of apoptosis. Furthermore, untargeted proteomics identified ribosomal proteins as additional cellular targets. Overall, these findings help to clarify the contribution of abundant EGCG oxidation products to the antiproliferative and HDAC modulating effects commonly attributed to the parent compound under cell culture conditions, underscoring the importance of investigating these oxidation products.

## 1. Introduction

Infusions prepared from the leaves of *Camellia sinensis* are among the most widely consumed beverages worldwide. In 2019, approximately five billion kilograms of tea were produced worldwide [1], making tea the second most consumed beverage after water. Tea consumption has been associated with a range of beneficial health effects, including cardioprotective, anti-obesity, and antidiabetic properties [2,3,4]. These effects are largely attributed to the wide variety of secondary metabolites present in the leaves, most notably polyphenols. Tea leaves contain up to 10% (*w*/*w*) catechins [5]. Earlier studies reported catechin contents of up to 30% of the dry mass [6]. Other functional constituents are theanine [7] and caffeine [8].

The most abundant catechin is epigallocatechin gallate (EGCG) [5]. Numerous in vitro and in vivo studies have investigated its bioactivity [9,10,11]. Mostly, the antioxidative properties have been suggested to be highly relevant for the beneficial effects [12]. However, although EGCG can protect other molecules from oxidative damage, its own catechol moiety is readily oxidized to a quinone, which is itself highly reactive and may promote Fenton chemistry, thereby increasing the formation of reactive oxygen species [13].

**Figure 1:**
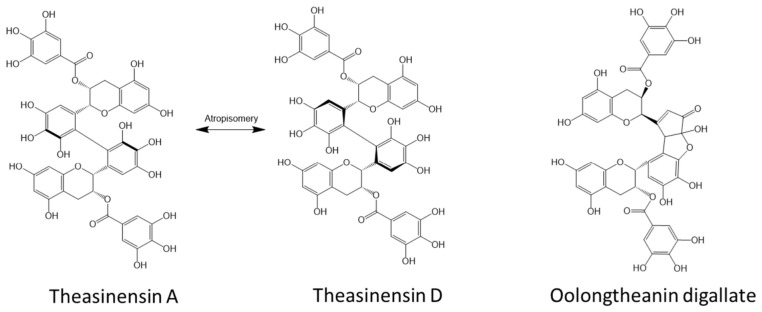
Chemical structure of the epigallocatechin gallate dimers used in this study, namely theasinensin A [TSA], theasinensin D [TSD] and oolongtheanin digallate [OTDG].

Like many other catechins, EGCG is unstable in aqueous solutions, with its stability strongly depending on pH, and it can be stabilized by ascorbic acid and other additives [14,15]. Because instability increases with both temperature and pH [15], it remains unclear whether certain bioactive properties are mediated by the parent compound EGCG or by its oxidation products. In a previous study, our group characterized three abundant dimers formed under physiological conditions [14]. The compounds investigated here are theasinensin A (TSA), theasinensin D (TSD), and oolongtheanin digallate (OTDG), as shown in Figure 1. Their formation was described by Tanaka and Kouno [15]. TSA, TSD, and OTDG are also present in green and black tea, as well as in their infusions, and their abundance increases with the degree of fermentation [16].

Antiproliferative effects and the onset of EGCG on several cellular mechanisms have been described [17]. These effects are attributed not only to its antioxidant and immunomodulatory properties and the induction of phase II metabolizing enzymes, but also to its specific interactions with individual carcinogens [17,18]. In addition, EGCG affects cell cycle progression and induces apoptotic signaling pathways, ultimately leading to tumor cell death. Both the transcription of apoptosis-associated genes and the activity of apoptotic enzymes have been reported to be altered by EGCG [19,20,21,22,23,24]. Known targets include cyclin-dependent kinases, several transcription factors and the tumor suppressor protein p53 [17,22,25,26,27].

Furthermore, the development and progression of cancer is strongly influenced by epigenetic mechanisms [28]. Among these, DNA methylation has received considerable attention, and the ability of catechins to modulate DNA methylation has been well documented [29,30,31]. Another fundamental epigenetic regulatory process is histone modification, which plays a critical role in the control of gene expression. Histones can undergo a wide range of amino acid side chain modifications [32], and these regulate gene expression through complex enzymatic cascades. Dysregulation of these processes can contribute to carcinogenesis [33]. One of the best-characterized histone modifications is lysine acetylation [34]. This balance is regulated by histone acetyltransferases and histone deacetylases (HDACs). HDACs are a recent target for cancer therapeutics [35], and specific inhibitors have shown anti-carcinogenic and anti-proliferative properties. Trichostatin A, for example, has already been evaluated in a clinical trial [36].

Whereas the cellular effects of EGCG on apoptosis and epigenetic activity have been intensively investigated, the contribution of EGCG oxidation products on this topic remains largely unknown. Therefore, this study examined the effects of TSA, TSD, and OTDG on key markers of apoptosis, HDAC gene expression and activity in HepG2 cells and compared them with those of the parent compound EGCG. Overall, this study addresses the extent to which these oxidation products contribute to the cellular effects commonly attributed to EGCG.

## 2. Results and Discussion

As described above, EGCG, theasinensins, and oolongtheanin digallate are rapidly converted into additional oxidation products under cell culture conditions. Therefore, all polyphenols were stabilized with ascorbic acid prior to incubation, which is a commonly used method [37,38]. Catalase was also added to the culture medium to prevent the accumulation of polyphenol-derived hydrogen peroxide. The effect of which is described here [39,40,41,42].

In all the results, either a one-way or a two-way Student’s *t*-test is applied to verify the statistical significance of the tested substance against the negative control (NC). This test does not allow for statistical evaluation between the test compounds and their used concentrations. When comparing these groups, we solely refer to the numerical value. Statistical significance is only claimed when mentioned specifically.

### 2.1. Apoptosis

#### 2.1.1. Apoptosis-Associated Gene Expression

Because antiproliferative and apoptotic effects of EGCG and TSA have previously been reported in the literature [43,44,45,46], the transcription of apoptosis-related genes was analyzed via quantitative reverse transcription PCR. Apoptosis can be initiated via either the extrinsic or the intrinsic pathway. In the extrinsic pathway, membrane receptors are activated by extracellular signals, leading to caspase-8 activation. The intrinsic pathway is triggered by intracellular stress signals and by an imbalance between pro- and anti-apoptotic members of the B-cell lymphoma 2 (Bcl-2) family, thereby activating the caspase-9 cascade. Both pathways lead to the activation of the effector caspases-3/7, which degrade cellular proteins such as lamin and actin, leading to cell death [47].

**Figure 2:**
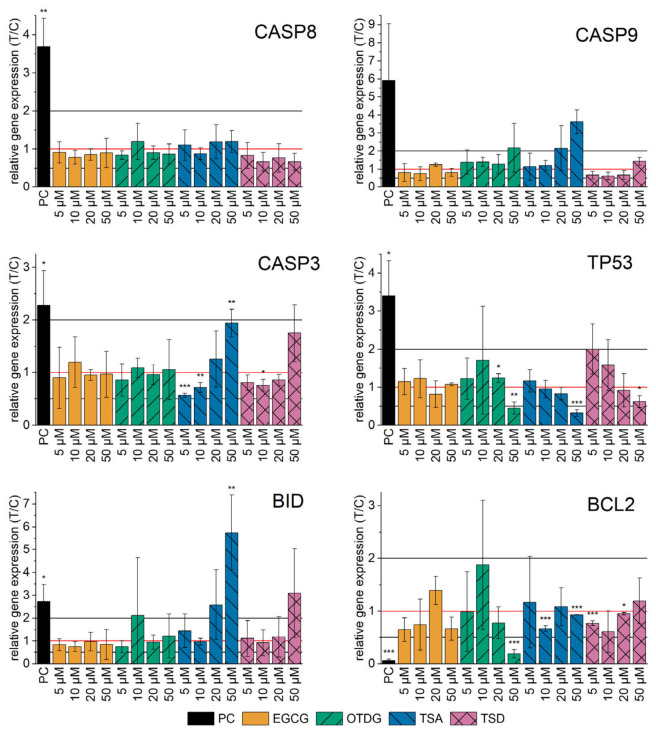
Transcription analysis of apoptosis-associated genes by quantitative reverse transcriptase polymerase chain reaction following incubation of HepG2 cells for 24 h with 5, 10, 20, or 50 µM epigallocatechin gallate (EGCG), oolongtheanin digallate (OTDG), theasinensin A (TSA), or theasinensin D (TSD), or with 1 µM camptothecin (CPT) as a positive control (PC). Relative values are expressed relative to the negative control (NC, red line), *n* ≥ 3, statistic evaluation via one-way *t*-test marked with asterisks: * *p* < 0.05, ** *p* < 0.01, *** *p* < 0.001.

Figure 2 shows relative transcription levels after 24 h of incubation with stabilized EGCG or its oxidation products OTDG, TSA, and TSD. Camptothecin (CPT) was used as a positive control to confirm the functionality of the transcriptional analysis. Only results considered biologically relevant were interpreted, defined here as those showing statistical significance relative to the negative control together with a fold change of <0.5 or >2.0.

Following treatment with 1 µM CPT, proapoptotic genes increased, generally exceeding a 2-fold change. In contrast, incubation with EGCG, theasinensins, or OTDG did not alter transcription of the extrinsic apoptosis-associated gene caspase-8 (*CASP8*) within the tested concentration range. Likewise, EGCG at concentrations between 5 and 50 µM did not significantly affect the transcription of the analyzed pro-apoptotic genes.

By contrast, its oxidation products—particularly the theasinensins—induced increased expression of genes associated with the intrinsic apoptotic pathway, includingcaspase-9 gene *CASP9* or BH3 interacting-domain death agonist (Bid) gene (*BID*). After incubation with 50 µM TSA, *CASP9* and *BID* transcription increased significantly to 3.6 and 5.7, respectively. TSD produced comparable but less pronounced effects; after treatment with 50 µM TSD, *BID* transcription increased to 3.1-fold. In addition, OTDG at the highest tested concentration caused a significant decrease in *TP53* transcription and a highly significant decrease in *BCL2* transcription. TSA also induced a concentration-dependent increase in caspase 3 (*CASP3*) transcription, suggesting downstream propagation of apoptotic signaling. The correlation between TSA incubation concentration and TP53 gene expression is inverse.

Apoptosis-related effects have been described in the literature for both EGCG and its major oxidation products, particularly the theasinensins. A variety of methods and experimental approaches is available to assess apoptotic interactions; however, transcriptional data following flavan-3-ol treatment remain limited. Although the present study did not reveal significant effects of EGCG on apoptosis-associated gene transcription in HepG2 cells, changes in other cell lines have been reported previously [19,20,21,23]. Hastak et al. [21], for example, described an altered transcription of genes encoding pro- and antiapoptotic proteins, including increased transcription of the Bcl-2-associated X protein (Bax) gene (*BAX*) and a decreased expression of *BCL2* [21,48]. In comparison, in the present study a significant reduction in *BCL2* transcription was observed only after OTDG treatment. Given the structural similarity between EGCG and its oxidation products, this OTDG-induced effect may arise from related cellular mechanisms. With respect to *BAX*, previous reports indicate functional interplay between the pro-apoptotic Bax protein and Bid, encoded by *BID*, which was also examined in the present study [49]. Accordingly, the reported increase in Bax abundance is consistent with the stimulatory effect of the theasinensins on *BID* transcription observed here.

#### 2.1.2. Caspase Activity

**Figure 3:**
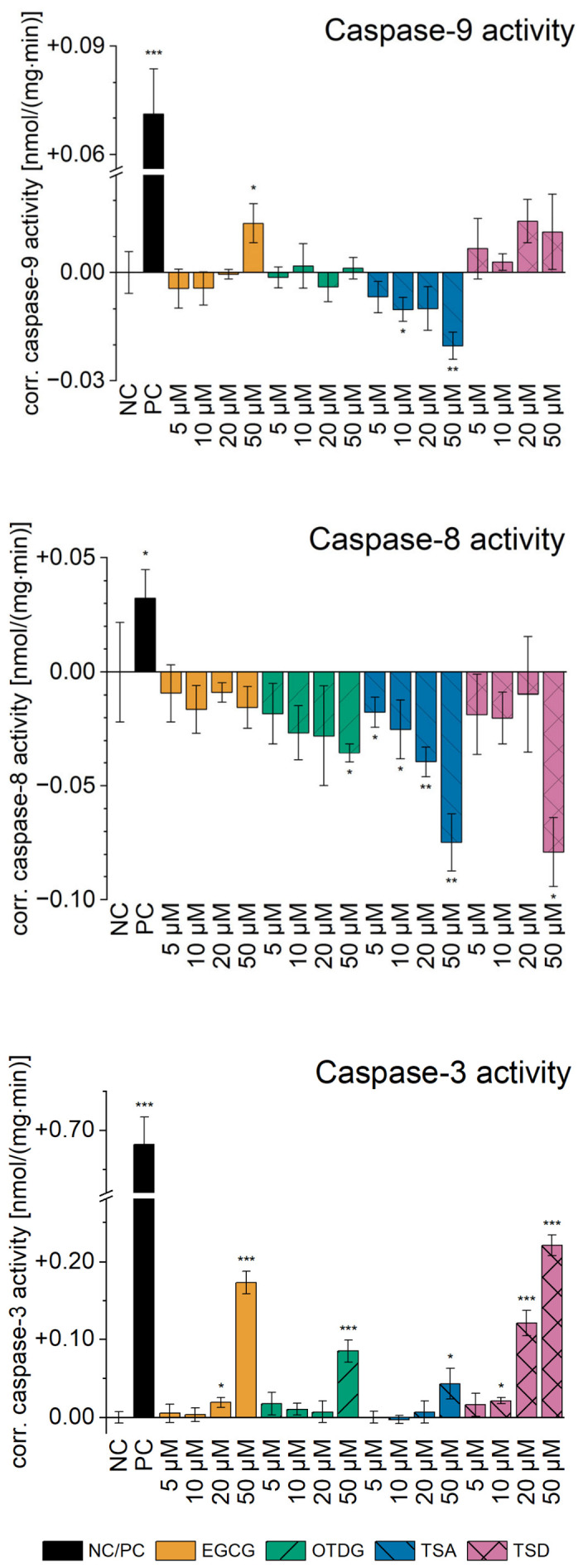
Enzyme activity of caspase-8, caspase-9, and caspase-3 after incubation of HepG2 cells for 24 h with 5, 10, 20, and 50 µM epigallocatechin gallate (EGCG), oolongtheanin digallate (OTDG), theasinensin A (TSA), and theasinensin D (TSD), or 2.5 µg/mL staurosporine (as caspase-8 positive control (PC)) or 10 µM camptothecin (as caspase-9 and caspase-3 PC). Absolute activity values were corrected for the negative control (NC) and normalized to the protein content of the cell lysate, *n* ≥ 3. Statistical significance was assessed using Student’s *t*-test versus the negative control: * *p* < 0.05, ** *p* < 0.01, *** *p* < 0.001.

Another important parameter for evaluating apoptosis induction by xenobiotics is the assessment of caspase activities. Members of this protease family are involved in both the extrinsic and intrinsic apoptotic pathways. Their enzymatic activity can be determined using specific fluorogenic substrates. Figure 3 shows the activities of the extrinsic initiator caspase-8, the intrinsic initiator caspase-9, and the effector caspase-3, each corrected for the respective negative control.

Activation of the effector caspase-3 leads to cleavage of poly(ADP-ribose) polymerase 1 (PARP1) and ultimately contributes to apoptotic cell death. Treatment with 50 µM of the tested flavan-3-ols resulted in a statistically significant increase in caspase-3 activity. The magnitude of this effect followed the order TSA < OTDG < EGCG < TSD. Among the EGCG dimers, the difference between TSA and TSD was particularly notable. At 50 µM, the highest concentration tested, corrected caspase-3 activities were 0.043 nmol/(mg·min) for TSA and 0.22 nmol/(mg·min) for TSD. Given the similar effects of these compounds on caspase-8 activity, differences in their effects on intrinsic caspase-9 activity may contribute to this discrepancy.

Comparison with the literature is limited here to EGCG and the theasinensins. Smith et al. [48] described an increase in caspase-3 activity in human LNCaP and DU-145 prostate adenocarcinoma cells after 10 µM incubation of EGCG for 24 h. According to the authors, this effect was associated with increased abundance of pro-apoptotic Bax family proteins. Although the present study was performed in HepG2 hepatocellular carcinoma cells, EGCG-mediated effects on caspase-3 and caspase-9 activity have also been reported for this cell model [22]. These findings are consistent with the statistically significant increase in both caspase-3 and caspase-9 activities observed in our study.

All tested flavan-3-ols (EGCG, OTDG, TSA, and TSD) reduced caspase-8 activity across the examined concentration range of 5–50 µM. For the oxidation products, this decrease was statistically significant at 50 µM. The atropisomers TSA and TSD showed with corrected activities of −0.075 nmol/(mg·min) and −0.079 nmol/(mg·min), respectively, whereas OTDG exerted a less pronounced effect (−0.036 nmol/(mg·min)). For TSA, all lower concentrations also resulted in significant changes in caspase-8 activity. To further confirm the findings, an additional protein expression validation by Western blot could be executed.

The effects on intrinsic caspase-9 activity differed among the tested flavan-3-ols. Incubation with TSA caused a highly significant decrease in caspase-9 activity to −0.020 nmol/(mg·min), whereas its atropisomer TSD increased caspase-9 activity, although this effect did not reach statistical significance. EGCG at 50 µM likewise increased caspase-9 activity, while OTDG had no detectable effect. In contrast to our findings, increased caspase-8 activity after 24 h of incubation with non-stabilized EGCG has been reported in human colon adenocarcinoma cells [50]. However, differences in the cell model, incubation conditions, compound stabilization, and treatment duration may account for this discrepancy. For TSA, Pan et al. [38] reported a 4.5-fold increase in caspase-9 activity after 3 h of incubation in U937 histiocytic lymphoma cells.

#### 2.1.3. Apoptotic Endpoint Analysis 

**Figure 4:**
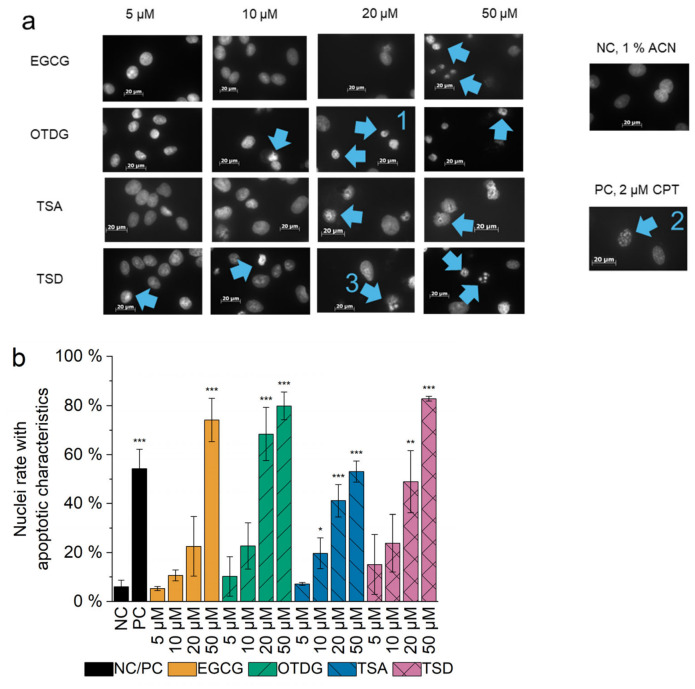
Apoptotic endpoint analysis via Hoechst-33342 fluorescence microscopy. (**a**) Representative fluorescence microscopy images of HepG2 cells after incubation, showing characteristic morphological changes at a magnification of 63 × 10. Arrow 1: reduced nuclear volume; arrow 2: nuclear fragmentation; arrow 3: chromatin condensation. (**b**) Percentage of nuclei exhibit apoptotic characteristics after incubation of HepG2 cells for 24 h with 5, 10, 20, or 50 µM epigallocatechin gallate (EGCG), oolongtheanin digallate (OTDG), theasinensin A (TSA), or theasinensin D (TSD), or with 2 µM camptothecin (CPT) as the positive control (PC). NC: negative control. *n* = 3. Statistical significance was assessed by two-way *t*-test and is indicated by asterisks: * *p* < 0.05, ** *p* < 0.01, *** *p* < 0.001.

Although the analyses of apoptosis-associated gene transcription and caspase activity indicate effects on apoptotic signaling pathways, additional regulatory mechanisms also contribute to apoptotic cell death. Therefore, morphological characteristics were included in the evaluation of the apoptotic effects of the flavan-3-ols. After staining with the DNA-binding dye Hoechst 33342, cells were visualized by fluorescence microscopy. The stained nuclei were examined for characteristic apoptotic features, particularly chromatin condensation, nuclear fragmentation, and reduced nuclear volume [44,51,52,53]. Figure 4 shows representative fluorescence microscopy images of stained HepG2 nuclei after incubation with the tested compounds at the indicated concentrations (Figure 4a), as well as the proportion of nuclei displaying apoptotic traits (Figure 4b).

After 24 h of incubation, the positive control (PC; 2 µM CPT), as well as all tested flavan-3-ols increased the proportion of nuclei exhibiting apoptotic morphology in a concentration-dependent manner. At 50 µM, TSD produced the highest proportion of apoptotic nuclei (83%), whereas EGCG, OTDG, and TSA yielded values of 74%, 80%, and 53%, respectively. Based on these findings, the apoptotic potential of the tested flavan-3-ols can be ranked as TSA < EGCG ≈ OTDG ≈TSD.

Using the same Hoechst 33342 fluorescence microscopy assay, Saeki et al. [44], demonstrated apoptosis-inducing effects of several flavan-3-ols in U937 cells. In agreement with our results, TSD was identified as the most potent apoptosis-inducing flavan-3-ol compared with EGCG and other theaflavins. Chromatin condensation was reported as a characteristic apoptotic alteration [44]. When considered together with the present Hoechst assay results, TSD also caused the strongest and most significant increase in caspase-3 activity. Given the role of caspase-3 as a key effector caspase in the apoptotic cascade, a relationship between elevated caspase-3 activity and the higher proportion of nuclei displaying apoptotic features appears plausible. To further investigate the apoptosis induction and determine the mechanism, more experiments, e.g., Poly (adenosine diphosphate-ribose) polymerase cleavage or Annexin V/P staining should be conducted.

### 2.2. HDAC Gene Expression and Activity

#### 2.2.1. Gene Expression Analysis of HDAC Isoforms

**Figure 5:**
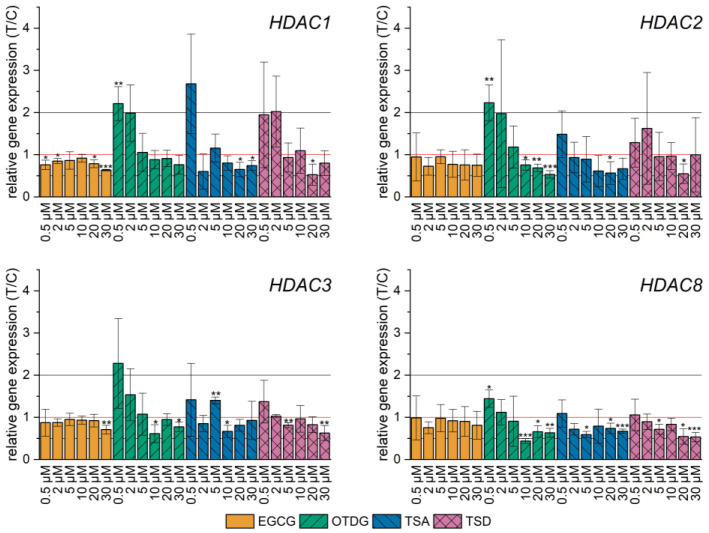
Relative gene expression of histone deacetylases (HDACs): *HDAC1*, *HDAC2*, *HDAC3*, and HDAC8, normalized to the housekeeping gene β-actin in HepG2 cells. Cells were incubated for 24 h with 0.5, 2, 5, 10, 20, or 30 µM theasinensin A (TSA), theasinensin D (TSD), oolongtheanin digallate (OTDG), or epigallocatechin gallate (EGCG). Relative values are expressed relative to the negative control (NC, red line), *n* = 3 × 3. Statistical significance was calculated using Student’s *t*-test compared with the negative control. Significance levels are indicated by asterisks: * *p* < 0.05, ** *p* < 0.01, *** *p* < 0.001.

The gene expression patterns of selected HDAC isoforms are determined in HepG2 cells by quantitative polymerase chain reaction (qPCR), relative to the housekeeping gene β-actin. The results are shown in Figure 5. As in the transcriptional analysis of apoptosis-associated genes, biological relevance was defined as a statistically significant difference from the NC combined with a fold change of <0.5 or >2.0.

Expression of *HDAC1* increased at lower incubation concentrations for all oxidation products. At higher concentrations, however, TSA, TSD, and EGCG led to a significant decrease in *HDAC1* expression. A similar pattern was observed for OTDG with respect to *HDAC2* expression. Significant changes in *HDAC2* expression were detected at all concentrations except 2 and 5 µM. In contrast, no significant changes were observed after EGCG treatment. TSA and TSD had little effect on *HDAC2* expression, except for a significant decrease at 20 µM. TSA and TSD affected *HDAC3* expression in a similar manner. Expression levels peaked at approximately 150% at 0.5 µM, although this increase did not reach statistical significance, and then decreased to around 75% at higher concentrations. OTDG showed the same general trend for *HDAC3* as observed for *HDAC1* and *HDAC2*.

However, for *HDAC3*, significant changes were observed only after incubation with 10 and 20 µM OTDG. EGCG did not markedly alter *HDAC3* expression, except for a decrease to approximately 80% at 30 µM. Among the analyzed isoforms, *HDAC8* expression was least affected by the tested polyphenols. EGCG did not significantly alter *HDAC8* expression. TSA and TSD reduced expression only moderately, to a minimum of approximately 60%. OTDG again followed the trend observed for the other isoforms, with increased expression at lower concentrations and decreased expression at higher concentrations, although its effect on *HDAC8* remained the weakest.

Overall, two findings deserve particular attention. First, under the conditions used in this study, EGCG did not significantly affect HDAC gene expression. In contrast, previous studies have reported reduced *HDAC1* expression after 24 h incubation with 10 µM EGCG in both monolayer cultures and spheroids [54]. In prostate cancer cells, incubation with 20 µM EGCG was also shown to reduce expression of class I HDACs. In another study, carcinogen-induced upregulation of gene expression in mice was normalized by administration of polyphenol-rich fruit extracts [55]. Second, the EGCG dimers showed an intriguing concentration-dependent pattern for *HDAC1*, *HDAC2*, and *HDAC3* expression. Elevated expression at low incubation concentrations shifted to reduced expression at higher concentrations. This trend was particularly pronounced for OTDG. TSA and TSD showed a similar tendency, although only *HDAC1* expression exceeded 200%. In general, the higher expression values were associated with relatively large standard deviations and therefore did not reach statistical significance compared with the negative control. It has been suggested that the half-life of EGCG and TSA is concentration-dependent, with lower concentrations showing reduced stability [56].

Such an inverse concentration-dependent bioactivity has only rarely been described in the literature. One possible explanation is the superposition of two distinct effects exerted by the dimers. At low concentrations, the polyphenols may interact with specific cellular targets and trigger signaling cascades that increase gene expression. At higher concentrations, however, the tested levels of 20 and 30 µM approach the IC_50_ value for cell viability [16], and cytotoxic effects—whether caused directly by chemical stress or by downstream signaling events—may induce a different cellular response. In addition, membrane fluidity is a known target of larger polyphenols, and these direct effects can be highly dependent on the nature of the substituent groups [57,58,59]. As these are hypotheses, further experiments would be needed to confirm them.

#### 2.2.2. HDAC Inhibition in Nuclear Extracts

**Figure 6:**
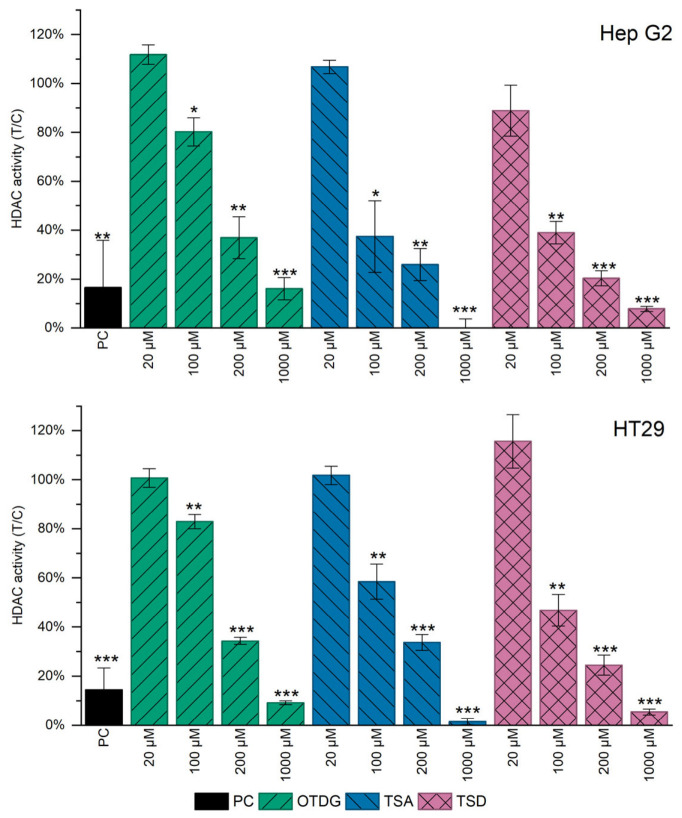
HDAC activity in nuclear protein extracts from HepG2 and HT29 cells, expressed as the ratio of treated samples to the negative control of the corresponding biological replicate, after incubation for 30 min with theasinensin A (TSA), theasinensin D (TSD), or oolongtheanin digallate (OTDG) at concentrations of 20, 100, 200, and 1000 µM. Trichostatin A (0.1 µM) was used as a positive control (PC). *n* = 3 × 3. Statistical significance was calculated using Student’s *t*-test versus the negative control. Significance levels are indicated by asterisks: * *p* < 0.05, ** *p* < 0.01, *** *p* < 0.001.

It has been reported that intracellular concentrations of approximately 20–1000 µM polyphenol dimers can be reached after cellular uptake [60]. Therefore, concentrations of 20, 100, 200, and 1000 µM were selected to assess direct inhibition of nuclear proteins. Trichostatin A, a well-established and highly selective HDAC inhibitor, was used as a positive control [61,62]. Nuclear protein fractions were isolated from three consecutive passages of human hepatoma HepG2 cells and human colon carcinoma HT29 cells. As shown in Supplementary Table S1, the absolute RFU values of HT29 nuclear extracts were higher than those of HepG2 extracts. HDAC activity was calculated as the ratio of the RFU of the polyphenol-treated protein extract to that of the negative control of the corresponding biological replicate. The resulting inhibition of HDAC activity is shown in Figure 6.

To date, strong HDAC-inhibitory activity has been reported for EGCG in HeLa nuclear extracts [63,64], whereas comparable studies on its oxidation products are lacking.

All tested compounds showed a concentration-dependent decline in HDAC activity using nuclear extracts. In both cell lines, 20 µM of the test compounds did not noticeably affect HDAC activity. At 100 µM, TSA exerted a stronger effect in HepG2 extracts, reducing HDAC activity to 40% of the control, whereas in HT29 nuclear extracts activity remained at 60%. At higher incubation concentrations, however, these differences were no longer apparent. TSD and OTDG showed comparable HDAC-inhibitory effects in HepG2 and HT29 nuclear extracts. The half-maximal inhibitory concentrations (IC_50_ values) are summarized in Table 1. TSA and OTDG exhibited higher IC_50_ values in HT29 nuclear extracts than in HepG2 nuclear extracts, corresponding to increases of 75% and 15%, respectively. In contrast, the IC_50_ value of TSD was essentially identical in both cell lines. In both models, OTDG showed the weakest inhibitory activity.

The three tested compounds inhibited HDAC activity in nuclear protein extracts from two different cell lines in a concentration-dependent manner. However, significant inhibition occurred only at relatively high micromolar concentrations, especially when compared with the specific HDAC inhibitor trichostatin A. This may indicate a rather unspecific reduction in HDAC activity, potentially caused by competitive enzyme interaction or partial protein denaturation related to the redox activity of the catechol groups. In pharmacology, polyphenols containing catechol structures are often discussed as pan-assay interference compounds (PAINS) [65]. Due to their potentially nonspecific enzyme-modulating properties, such compounds may yield positive signals in a wide range of enzyme-based assays.

The calculated IC_50_ values suggest that TSA exerts stronger HDAC-inhibitory effects in HepG2 nuclear extracts than in HT29 nuclear extracts. However, it should be noted that the absolute AMC signal was higher in HT29 nuclear extracts. In addition, OTDG appeared less active overall than the theasinensins.

A previous study reported that 50 µM EGCG reduced HDAC activity in HeLa nuclear extracts by approximately 50% [64]. Furthermore, molecular docking experiments suggested potential binding of EGCG to HDAC2, HDAC3, HDAC4, and HDAC7, consistent with competitive inhibition of these enzymes. These binding sites may also represent possible targets for EGCG dimers. The calculated IC_50_ values of TSA and TSD, ranging from approximately 60 to 80 µM, are within the same order of magnitude as values reported in the literature for EGCG [64]. As already mentioned, EGCG is quite unstable in aqueous cell culture conditions [14]. Although the cited literature provides valuable insight into the epigenetic activity of EGCG, it does not account for this instability. Since no information is given regarding incubation time or buffer pH in the nuclear extract assay, formation of EGCG oxidation products may have contributed to the reported inhibition of HDAC by EGCG. The biological relevance of the results should be regarded with caution since the concentration range applied in this assay is unlikely to be found in vivo.

#### 2.2.3. HDAC Inhibition in HepG2 Cells

**Figure 7:**
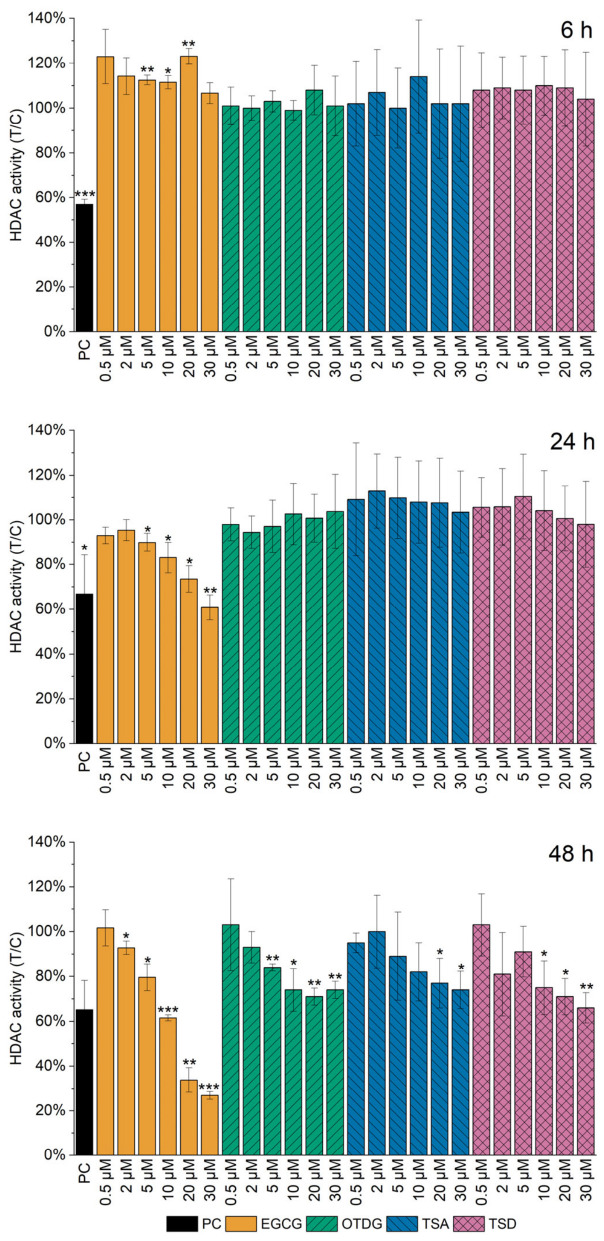
Histone deacetylase (HDAC) activity in HepG2 cells, expressed as the ratio of treated samples to the negative control of the corresponding biological replicate, after incubation with theasinensin A (TSA), theasinensin D (TSD), oolongtheanin digallate (OTDG), or epigallocatechin gallate (EGCG) at concentrations of 0.5, 2, 5, 10, 20, and 30 µM for 6, 24, and 48 h. Trichostatin A (0.1 µM) served as a positive control (PC), *n* = 3 × 3. Statistical significance was calculated using Student’s *t*-test versus the negative control. Significance levels are indicated by asterisks: * *p* < 0.05, ** *p* < 0.01, *** *p* < 0.001.

Because HepG2 nuclear extracts showed a stronger response to EGCG dimers than HT29 nuclear extracts, HepG2 cells were selected for the subsequent cell-based experiments. Direct exposure of HepG2 cells to the dimers did not result in a pronounced concentration-dependent inhibition of HDAC activity after 6 or 24 h of incubation (Figure 7). At 6 h, EGCG even increased HDAC activity. However, after 48 h, EGCG showed a concentration-dependent reduction in cellular HDAC activity, reaching 60% of the negative control, a level comparable to that observed for the positive control.

After 48 h of incubation, TSD and TSA significantly reduced HDAC activity at 10 and 20 µM. OTDG significantly lowered HDAC activity at 5 µM, while EGCG did so already at 2 µM. At higher concentrations, theasinensins and OTDG produced a concentration-dependent decline in HDAC activity to approximately 75% of the negative control. In contrast, EGCG reduced HDAC activity to 25% at 30 µM. Thus, EGCG showed a markedly stronger effect on cellular HDAC activity than its dimeric oxidation products.

Khan et al. [26] incubated HeLa cells with 25 µM of EGCG and determined the HDAC activity in the nuclear extracts. After 48 h, the HDAC activity remained at 86% of the control. In another study using HT29 cells, direct incubation with 50 µM EGCG for 24 h did not significantly reduce HDAC activity, whereas 100 µM EGCG decreased activity by approximately 50% [66]. In the cited studies [26,66], EGCG was not stabilized, making rapid degradation and oxidation under cell culture conditions likely. Formation of oxidation products may have contributed to the comparatively weaker HDAC inhibition reported in those studies. However, because TSA, TSD, and OTDG showed only limited effects on HDAC activity in our cell-based assay, the present results rather suggest that EGCG itself is a more potent HDAC inhibitor than its major dimeric oxidation products. Other polyphenols, such as the soy isoflavone genistein and chlorogenic acid, have also been described as HDAC inhibitors, but with relatively high IC_50_ values of 100 µM and 250 µM, respectively [66].

Overall, it should be emphasized that effects on enzyme activity should ideally be assessed at concentrations that do not cause substantial cytotoxicity, in order to avoid nonspecific effects. In HepG2 cells, EGCG cytotoxic IC_50_ values between 100 and 218 µM have been reported [67,68]. In this study, EGCG significantly inhibits HDAC activity at concentrations up to 30 µM, which are below the reported cytotoxic range, suggesting that nonspecific cytotoxic effects are unlikely to explain the observed inhibition. However, HepG2 cell viability was reduced by 50% after incubation with 30 µM TSA or TSD, or 70 µM OTDG, for 24 h. This suggests that, in the case of the oxidation products, HDAC inhibition may partly be associated with reduced cell viability.

However, when discussing the results, one should take into consideration that no direct assessment of histone acetylation or chromatin remodeling was performed.

### 2.3. Proteomics Approach

To further validate the findings from the gene expression analysis, a proteomics approach was performed in HepG2 cells according to Keuter et al. [69]. Cells were treated with sub-cytotoxic concentrations (0.5, 2, and 20 µM) to avoid cytotoxic side effects. Proteins were extracted, digested with trypsin, and analyzed by HPLC coupled to high-resolution mass spectrometry (HRMS). Protein identification and label-free quantification (LFQ) were carried out by comparison with a human proteome database using the open-source software MaxQuant [70]. Based on LFQ-values, *p*-values, and fold changes were calculated and analyzed using the STRING Network [71]. By integrating information from databases containing annotated protein functions and localization data, biological effects and cellular compartments associated with the detected protein changes could be assigned. These associations are expressed as enrichment scores (ES). For the tested compounds, significant hits were found in the Gene Ontology [72] database categories *Cellular* Component and Biological Process as well as in the Reactome Pathways [73] database.

For TSA, only incubation with 2 µM produced proteomic alterations sufficient to yield enriched clusters, whereas for TSD this was observed only at 20 µM. OTDG was the only compound that modulated the proteome of HepG2 cells at all tested concentrations. Selected enrichment scores are shown in Table 2. It should be noted that all listed pathway hits were derived from the Reactome Pathway database [73].

In the Cellular Component category of the Gene Ontology database [72], all enriched clusters were related to the ribosome. TSD and OTDG showed identical enrichment scores, although this occurred at 20 µM for TSD and at 0.5 µM for OTDG. For OTDG, enrichment scores decreased with increasing concentration. TSA showed the weakest effects in this category and did not yield hits for the clusters large ribosomal subunit or ribosomal subunit. A similar pattern was observed in the Biological Process category, where TSD and OTDG showed the highest enrichment scores for cytoplasmic translation at 20 µM and 0.5 µM, respectively. Again, OTDG showed an inverse concentration-dependent decline in enrichment score. Hits in the Reactome Pathway database [73] were observed only for OTDG. These enriched clusters primarily involved cell-surface- and membrane-associated proteins. After incubation with 2 and 20 µM OTDG, fibrinogen and intercellular adhesion molecule were among the most strongly upregulated proteins. In addition, incubation with 2 µM OTDG enriched the apoptosis-induced DNA fragmentation cluster, mainly due to reduced abundance of histone H1 proteins.

To the best of our knowledge, this is the first report describing the effects of TSA, TSD, and OTDG on the proteome of a cell line. For TSD and OTDG, the observed changes in protein abundance may be related to cell cycle arrest, a mechanism frequently described in the literature for several classes of polyphenols [74,75,76]. Zhao et al. [77] investigated the effects of green and black tea extracts on cell cycle progression and apoptosis in the human gastric cancer cell line SGC-7901. The authors reported a stronger cell cycle arrest after treatment with the fermented black tea extract, suggesting that oxidized polyphenols may contribute to this effect. Nevertheless, it remains unclear why TSA affected cellular targets only weakly compared with the other dimeric oxidation products, TSD and OTDG. However, a previous study reported differences in intracellular concentrations among these three oxidation products, which may contribute to the effects observed here [60].

## 3. Conclusions

Combining the data from all parts of the study allows the following conclusions to be drawn for HepG2 cells under the selected in vitro conditions.

EGCG did not significantly influence the expression of the analyzed apoptosis-associated genes. However, at the highest tested concentration, it increased caspase-9 and caspase-3 activity. In agreement with these findings, a significant induction of apoptosis was detected only at this concentration. EGCG also inhibited HDAC activity in HepG2 cells. This effect is unlikely to be explained by direct inhibition alone, since the intracellular concentrations achieved under the tested conditions would probably not be sufficient to account for the observed magnitude of inhibition. In contrast to the results obtained with nuclear extracts, no corresponding decrease in HDAC activity attributable solely to direct interaction was observed in intact HepG2 cells. Previous studies [26,66] have described EGCG as only a weak inhibitor of HDAC activity in cell-based systems. However, this conclusion may potentially underestimate the contribution of the oxidation products, as these studies did not stabilize EGCG during incubation, thereby allowing its conversion into oxidation products with lower HDAC-inhibitory potency. Consistent with this interpretation, EGCG caused only minor or no changes in the expression of class I HDACs.

Among the tested compounds, TSA showed the lowest potential to induce apoptosis in HepG2 cells, which is in line with its comparatively weak effects on caspase-8, caspase-9, and caspase-3 activity. Nevertheless, TSA did affect the expression of both pro-apoptotic genes, including *CASP9*, *CASP3*, and *TP53*, indicating that modulation of gene expression is not necessarily directly reflected at the level of enzyme activity. TSA reduced HDAC activity only in the assays using nuclear extract, whereas no significant effect was detected after direct incubation with intact cells. This does not fully agree with the gene expression results, where higher concentrations reduced expression of several HDAC isoforms, particularly *HDAC8*. Overall, the influence of TSA on the HepG2 proteome was limited under the applied conditions.

As the atropisomer of TSA, TSD might be expected to exert similar effects. However, no major changes in pro-apoptotic gene expression were observed. In addition, the activities of initiator caspases-8 and -9 remained largely unaffected. In contrast, the activity of the effector caspase-3 increased markedly and in a concentration-dependent manner after TSD treatment, which is consistent with the increased proportion of apoptotic cells detected morphologically. At the same time, the effects of TSD on HDAC activity, *HDAC* gene expression, and the proteome were broadly comparable to those of TSA.

OTDG affected the investigated cellular targets differently from the two theasinensins. Transcriptional analysis revealed downregulation of *TP53* and *BCL2*, whereas the activities of initiator caspases-8 and -9 were not markedly altered. Caspase-3 activity was significantly increased only at the highest tested concentration. This finding does not fully correspond to the Hoechst assay results, in which apoptotic features were already strongly increased at concentrations above 20 µM. In addition, lower incubation concentrations of OTDG exerted stronger effects on HDAC gene expression and on the HepG2 proteome than higher concentrations.

Taken together, these data indicate that the parent compound EGCG and its oxidation products differ markedly in their effects on cellular targets involved in apoptosis induction and HDAC-related cellular regulation in HepG2 cells. Because EGCG is chemically unstable under cell culture conditions and is also transformed during tea manufacturing, the contribution of its oxidation products should be taken into account when interpreting in vitro data. In particular, stabilization of EGCG during in vitro experiments is essential in order to avoid misleading or artificial results. However, because the present study was limited to an in vitro HepG2 model and did not include primary cells, in vivo experiments, or pharmacokinetic analyses, the mechanisms of apoptotic induction and epigenetic relevance of these findings require further validation.

## 4. Materials and Methods

Further methodological details are provided in the Supplementary Materials.

### 4.1. Chemicals and Reagents

Pure water was generated via reverse osmosis using a miniRO station (Veolia Water Solutions & Technologies ELGA GmbH, Celle, Germany).

The following chemicals and reagents were used: 1,4-diazabicyclo[2.2.2]octane (DABCO; Carl Roth, Karlsruhe, Germany), 7-amino-4-trifluoromethylcoumarin (AFC; Merck KGaA, Darmstadt, Germany), Ac-DEVD-AFC (Cayman Chemical, Ann Arbor, MI, USA), Ac-IETD-AFC (Cayman Chemical), Ac-LEHD-AFC (Cayman Chemical), acetonitrile (ACN, LC-MS grade; Fisher Scientific GmbH, Schwerte, Germany), apoptosis-associated gene primers (RealTimePrimers LLC, Elkins Park, PA, USA; and QIAGEN N.V., Venlo, The Netherlands), Boc-Lys(Ac)-AMC and Boc-Lys-AMC (Bachem AG, Bubendorf, Switzerland), calcium chloride (Carl Roth), camptothecin (CPT; Cayman Chemical), catalase from bovine liver (2000–5000 U/mg protein; Sigma-Aldrich Chemie GmbH, Taufkirchen, Germany), iScript™ cDNA Synthesis Kit (Bio-Rad Laboratories GmbH, Munich, Germany), CHAPS (Carl Roth), citric acid (Grüssing GmbH, Filsum, Germany), copper(II) sulfate pentahydrate (Carl Roth), dimethyl sulfoxide (DMSO, 99.5%; Carl Roth), disodium phosphate (Carl Roth), dithiothreitol (DTT; Carl Roth), Dulbecco’s modified Eagle’s medium (DMEM; Fisher Scientific), EDTA and Na-EDTA (Carl Roth), epigallocatechin gallate (EGCG, >99%; Extrasynthese, Genay, France), ethanol (absolute; Fisher Scientific), fetal calf serum (FCS; Fisher Scientific), glycerol (Sigma-Aldrich), HDAC primers (Biolegio, Nijmegen, The Netherlands), HEPES (Carl Roth), Hoechst 33342 (Cayman Chemical), iTaq Universal SYBR Green Supermix (Bio-Rad), L-ascorbic acid (Carl Roth), Mowiol 4-88 (Carl Roth), nicotinamide adenine dinucleotide, reduced form (NADH; Carl Roth), penicillin/streptomycin solution (1:1, *v*/*v*; 10,000 U/mL and 10,000 µg/mL, respectively; Fisher Scientific), peqGOLD Total RNA Kit (VWR International), PIPES (Carl Roth), potassium hydrogen phosphate (Carl Roth), pyruvic acid (≥98%; Carl Roth), sodium chloride (Carl Roth), staurosporine (STS; Cayman Chemical), Triton X-100 (Carl Roth), TRIS and TRIS-HCl (VWR International GmbH, Darmstadt, Germany), trichostatin A (TCI Deutschland GmbH, Eschborn, Germany), trypsin solution (0.05%, *w*/*v*; Fisher Scientific), tripotassium phosphate (≥98%; Sigma-Aldrich), and Tween 20 (Carl Roth).

Oolongtheanin digallate, theasinensin A, and theasinensin D were isolated and structurally elucidated as previously reported [14].

### 4.2. Software and Statistical Analysis

OriginPro 2026 and Microsoft Excel were used for data processing and statistical evaluation. For statistical analysis, Student’s *t*-tests were applied to compare test samples with the corresponding negative controls of each assay. Significance levels are indicated in the figures as follows: * *p* < 0.05, ** *p* < 0.01, *** *p* < 0.001.

IC_50_ values were calculated by fitting the sigmoidal DoseResp function to the experimental data. Peptide identification and calculation of LFQ values from HRMS data were performed using MaxQuant 2.5.1 [70]. Proteomic cluster analysis was conducted with STRING [71] version 12.0.

### 4.3. Cell Culture

HepG2 hepatocellular carcinoma cells (DSMZ, German Collection of Microorganisms and Cell Cultures GmbH, Braunschweig, Germany; ACC 180) and HT29 colon adenocarcinoma cells (DSMZ; ACC 299) were used. Both cell lines were cultured at 37 °C in a humidified atmosphere containing 5% CO_2_ in Dulbecco’s modified Eagle’s medium (DMEM) supplemented with penicillin, streptomycin, and 10% fetal calf serum (FCS).

For the different assays, cells were seeded into culture vessels of various formats. The corresponding cell numbers, vessels, and growth times are listed in Table 3. Cells were grown for 48 h to reach approximately 70–80% confluence unless stated otherwise. The culture medium was then replaced with FCS-free DMEM.

For incubation with polyphenols, acetonitrile (ACN) or dimethyl sulfoxide (DMSO) was used as solvent and diluted 1:99 in FCS-free DMEM. In addition, all samples, solvent controls, and positive controls were supplemented with stabilization solution containing 1 mM ascorbic acid and 500 U/mL catalase. Nuclear protein fractions used for HDAC inhibition assays were prepared from cell suspensions remaining after subcultivation.

### 4.4. Caspase Activity Determination

Cells were incubated with polyphenols or positive controls for 24 h. After incubation, cells were washed and lysed with Triton X-100. Protein concentrations in the lysates were determined using the bicinchoninic acid (BCA) assay for normalization. Caspase activity was measured by incubating the lysates with the respective fluorogenic caspase substrates and quantifying the cleavage products using a Tecan plate reader. Results are reported as absolute activity values and were corrected by subtraction of the negative control. Further protocol details are provided in the Supplementary Materials (Section S2.1).

### 4.5. Hoechst-33342 Fluorescence Microscopy

Cells were incubated for 24 h with the test compounds (5–50 µM) or with CPT (2 µM) as positive control. Afterwards, the cells were washed and fixed with ice-cold methanol for 1 h at −20 °C. Slides were then dried at room temperature. Cells were stained with Hoechst 33342 solution and subsequently washed with copper(II) sulfate solution. After an additional washing step with PBS, the stain was mounted and stabilized with Mowiol solution. Nuclei were visualized by fluorescence microscopy at an excitation wavelength of 358 nm and an emission wavelength of 460 nm. For each slide, 1000 nuclei were evaluated. Further protocol details are provided in the Supplementary Materials (Section S2.2).

### 4.6. HDAC Inhibition

The HDAC inhibition assay in nuclear extracts was performed according to the protocol described by Reddy et al. [78]. Protein extracts, prepared by the protocol of Waldecker et al. [79], were incubated with assay buffer, reaction solution, and inhibitor for 30 min. Subsequently, stop solution was added, and the secondary reaction was initiated by addition of developer solution followed by incubation for a further 15 min. Fluorescence was measured with a Tecan plate reader at an excitation wavelength of 360 nm and an emission wavelength of 460 nm. Trichostatin A, whose efficacy was demonstrated by Chiba et al. [80], served as a PC.

For determination of HDAC activity in intact cells, HepG2 cells were incubated with polyphenols or positive control for 6, 24, or 48 h. After washing, cells were incubated with a mixture of medium, assay buffer, and substrate for 30 min. Developer solution was then added, and fluorescence was measured as described above. Further protocol details are provided in the Supplementary Materials (Sections S2.3 and S2.4). The pipetting scheme is given in Supplementary Table S3.

### 4.7. Primer Design

Primers for HDAC gene expression analysis were designed using the Primer-BLAST tool provided by the NCBI [81]. Annealing temperatures were optimized to obtain the lowest possible quantification cycle (Cq) values. Further details are provided in the Supplementary Materials (Section S2.5). Primer sequences are listed in Supplementary Table S4.

### 4.8. Gene Expression Analysis

Cells were incubated with the polyphenols for 24 h before RNA extraction using the *peqGOLD kit*. RNA concentration was determined with a NanoDrop 1000 spectrophotometer. cDNA was synthesized using the iScripts cDNA Synthesis Kit. Quantitative PCR (qPCR) was performed with the iTaq Universal SYBR Green Supermix and the respective primers. Relative transcription levels were calculated using the ^ΔΔ^Cq method. The same approach was used for the HDAC genes with self-designed primers. Further details are provided in the Supplementary Materials (Sections S2.5 and S2.6). Thermocycling programs are listed in Supplementary Tables S5 and S6.

### 4.9. Proteomics

Proteomic analysis was performed according to the protocol described by Keuter et al. [69]. Cells were cultured in 6-well plates and incubated for 24 h. After incubation, the medium was removed and the cells were washed with ice-cold PBS. Cells were then scraped into microcentrifuge tubes and centrifuged at 860× *g* for 5 min at 4 °C. The supernatant was discarded, and the pellet was lysed using filter-aided sample preparation (FASP) lysis buffer [4] followed by sonication with an ultrasonic probe for 10 s at 3 W. The samples were then shaken and heated to 95 °C for 5 min. Cell lysates were centrifuged at 16,000× *g* for 5 min, and protein content was determined using the tryptophan assay. For FASP digestion, the filter unit was loaded with 200 µL urea buffer and approximately 300 µg protein, followed by centrifugation at 14,000× *g* for 10 min. Proteins on the filter were then treated with 100 µL 50 mM iodoacetamide for 20 min. After centrifugation at 14,000× *g* for 10 min, the filter was washed three times with 100 µL urea buffer and subsequently three times with ammonium bicarbonate (ABC) buffer. After each washing step, the filter was centrifuged at 14,000× *g* for 15 min. Next, 40 µL ABC buffer and 15 µL trypsin solution (0.2 µg/µL) were added to the filter. Proteins were digested for at least 20 h at 37 °C with shaking. Peptides were recovered by centrifugation at 14,000× *g* for 10 min, and the filter was washed twice with 40 µL ABC buffer. To the combined eluates, 2 µL formic acid (FA) was added. Before analysis, peptides were desalted by solid-phase extraction (SPE) using a Strata-X 33 µm RP 30 mg/1 mL cartridge. The cartridge was conditioned with methanol and equilibrated with water containing 1% FA. The sample was then loaded onto the cartridge and washed with water containing 1% FA. Peptides were eluted with 1% FA in methanol/water (9:1, *v*/*v*). The solvent was evaporated completely, and the residue was reconstituted in 100 µL ACN/water (1:9, *v*/*v*). HPLC and HRMS conditions are listed in detail in the publication of Keuter et al. [4].

## Figures and Tables

**Table 1:** IC_50_ values of theasinensin A (TSA), theasinensin D (TSD), and oolongtheanin digallate (OTDG) for histone deacetylase inhibition in nuclear extracts from HepG2 and HT29 cells.

Cell Line	Compound	IC_50_ Value [µM]
HepG2	TSA	69
	TSD	74
	OTDG	130
HT29	TSA	121
	TSD	73
	OTDG	149

**Table 2:** Selected enrichment scores of the clusters from the Gene Ontology [72] and the Reactome Pathway [73] databases as determined using STRING [71] version 12.0. Up- and down-regulation of the clusters marked by ↓ and ↑. Protein samples of HepG2 cells were incubated with 2 µM of theasinensin A (TSA), 20 µM of theasinensin D (TSD), or 0.5, 2, and 20 µM oolongtheanin digallate (OTDG) for 24 h. Protein quali- and quantification were performed using MaxQuant [70].

Database	Cluster Description	TSA 2 µM	TSD 20 µM	OTDG 0.5 µM	OTDG 2 µM	OTDG 20 µM
Gene Ontology (Cellular Component)	Cytosolic large ribosomal subunit ↓	0.58	1.39	1.38	1.17	0.96
	Large ribosomal subunit ↓	-	1.29	1.29	1.08	0.91
	Cytosolic ribosome ↓	0.36	1.27	1.27	0.96	0.82
	Ribosomal subunit ↓	-	-	1.22	0.91	0.80
Gene Ontology (Biological Process)	Cytoplasmic translation ↓	-	1.07	1.07	0.85	0.66
Reactome Pathways	Integrin cell surface interactions ↑	-	-	-	2.19	3.95
	Extracellular matrix organization ↑	-	-	2.04	-	2.14
	Apoptosis-induced DNA fragmentation ↓	-	-	-	3.00	-

**Table 3:** Cell count, dishes, and growth time used for the corresponding in vitro assay.

Assay	Cell Number	Cell Culture Vessels	Growth Time [h]
Apoptosis-associated gene expression	900,000/dish	60 mm cell culture dish	48
Caspase activity	900,000/dish	60 mm cell culture dish	48
Hoechst-33342 fluorescence microscopy	1,000,000/dish	QuadriPerm^®^ coated with poly-L-Lysine	24
HDAC nuclear extracts	1,000,000/flask	75 cm^2^ Flask	96
HDAC activity	10,000/well	96-well plate	48
HDAC gene expression	300,000/well	6-well plate	48

## Data Availability

The data presented in this study are available on request from the corresponding authors.

## Supplementary Materials

The following supporting information can be downloaded at https://www.mdpi.com/article/10.3390/molecules31122101/s1, Section S1 HDAC Activity including Table S1: RFU values of the PC and NC. Section S2 Methods contains S2.1 Caspase Activity with Table S2: Pipetting scheme, S2.2 Hoechst 33342 Fluorescence Microscopy, S2.3 Preparation of nuclear HT29 and HepG2 cell extracts, S2.4 HDAC Inhibition with Table S3: Pipetting scheme nuclear HDAC inhibition, S2.5 HDAC gene expression with Tables S4: Primer validation data and Table S5: qPCR parameters and S2.6 Apoptosis-associated gene expression with Table S6: qPCR parameters.

## Abbreviations

ACN	Acetonitrile
*BAX*	Bcl-2-associated X protein gene
Bax	Bcl-2-associated X protein
*BCL2*	B-cell lymphoma 2 gene
Bcl-2	B-cell lymphoma 2
*BID*	BH3 interacting-domain death agonist gene
Bid	BH3 interacting-domain death agonist
CPT	Camptothecin
*CASP3*	Caspase-3 gene
*CASP8*	Caspase-8 gene
*CASP9*	Caspase-9 gene
Cq	Quantification cycle
DMSO	Dimethyl sulfoxide
EGCG	Epigallocatechin gallate
ES	Enrichment score
FCS or FBS	choose one and use consistently
HDAC	Histone deacetylase
HPLC	High-performance liquid chromatography
HRMS	High-resolution mass spectrometry
IC_50_	Half-maximal inhibitory concentration
NC	Negative control
OTDG	Oolongtheanin digallate
PC	Positive control
qPCR	Quantitative polymerase chain reaction
*TP53*	Tumor protein p53 gene
TSA	Theasinensin A
TSD	Theasinensin D
