# Supplementary material for: Changes in HDAC Expression and Activity by Oolongtheanin Digallate and Theasinensins and the Onset of Apoptosis
Source: Molecules. 2026 Jun 15;31(12):2101. doi: 10.3390/molecules31122101 (PMC13304866; doi:10.3390/molecules31122101)
Supplement: Supplementary file 1 [file molecules-31-02101-s001.zip › molecules-4355056-supplementary.pdf]

Supplementary Material

Changes in HDAC Expression and Activity by Oolongtheanin Digallate and Theasinensins and the Onset of Apoptosis

Johannes Gröne, Julian Alfke, Marco Fortmann, Uta Kampermann, Mustafa Masoodi, Hans-Ulrich Humpf and Melanie Esselen*

Institute of Food Chemistry, University of Münster, Corrensstr. 45, 48149 Münster, Germany
*Correspondence: esselen@uni-muenster.de;

Table of Contents

Section S1 HDAC Activity

- **Table S1**: RFU values of the positive control (PC) and the negative control (NC)

Section S2 Methods

S2.1 Caspase Activity

- **Table S2:** Pipetting scheme

S2.2 Hoechst 33342 Fluorescence Microscopy

S2.3 Preparation of nuclear HT29 and HepG2 cell extracts

S2.4 HDAC Inhibition

- **Table S3**: Pipetting scheme nuclear HDAC inhibition

S2.5 HDAC gene expression

- **Table S4**: Primer validation data
- **Table S5**: qPCR parameters

S2.6 Apoptosis-associated gene expression

- **Table S6:** qPCR parameters

References

S1. HDAC Activity

Table S1: RFU values of the positive control (PC) and the negative control (NC) determined with the HDAC activity assay using HT29 and HepG2 cells in three biological replicates (BR)

Sample [RFU]	HepG2 cells	HT29 cells
PC BR1	9303	22948
PC BR2	9166	22805
PC BR3	20434	30731
NC BR1	3228	5323
NC BR2	2893	3550
NC BR3	4172	8039

S2. Methods

S2.1 Caspase activity

Incubation solutions were prepared by diluting the stock solutions 1:99 (v/v) in serum-free DMEM. Test concentrations were 5, 10, 20, and 50 µM. Acetonitrile (ACN, 1%) was used as the negative control, whereas 10 µM camptothecin (CPT) and 2.5 µg/mL staurosporine (STS) served as positive controls. Cells were incubated for 24 h. After incubation, cells were washed with 1 mL PBS prewarmed to 37 °C. Following removal of PBS, the culture dishes were placed on ice, and 600 µL lysis buffer was added. The lysis buffer consisted of 121.14 mg TRIS, 584.4 mg sodium chloride, 29.224 mg EDTA, and 1 mL Triton X-100, adjusted to 100 mL with ultrapure water at pH 7.4. After 15 min of lysis, cells were scraped off and transferred to microcentrifuge tubes. The lysates were centrifuged at 10,000 × g for 10 min at 4 °C, and 300 µL of the supernatant was transferred to a fresh tube. These lysates were used for both protein quantification and caspase activity assays. Protein concentrations were determined using the bicinchoninic acid (BCA) assay. Cell lysates were diluted 1:4 (v/v) with lysis buffer, and 15 µL of each sample, as well as blank samples (lysis buffer) and calibration standards, were pipetted in triplicate into a 96-well plate. For calibration, bovine serum albumin (BSA) solutions in lysis buffer were prepared in a concentration range of 60–1000 µg/mL. Subsequently, 200 µL of reagent solution was added to each well. The reagent solution consisted of 98% (v/v) Pierce™ BCA Protein Assay Reagent A and 2% (v/v) of a freshly prepared 6.25% (w/v) copper(II) sulfate pentahydrate solution in ultrapure water. The plate was covered and incubated for 30 min at 37 °C. Absorbance was then measured at 560 nm using an Infinite 200 PRO plate reader (Tecan Group Ltd., Männedorf, Switzerland). Protein concentrations were calculated by linear regression using the BSA calibration curve.

Caspase activity measurements were performed in a black 96-well plate. An AFC calibration series in lysis buffer was prepared at concentrations of 1.25, 2.5, 5, 10, and 50 µM. For the assay, 5× reaction buffer and AFC substrate solutions were prepared. The 5× reaction buffer consisted of 1510 mg PIPES, 500 mg CHAPS, and 372 mg Na-EDTA, adjusted to 100 mL with ultrapure water at pH 7.4. Different AFC-labeled peptide substrates were used for the individual caspases: Ac-IETD-AFC for caspase-8, Ac-LEHD-AFC for caspase-9, and Ac-DEVD-AFC for caspase-3. The reaction solution (Table S2) contained 20% (v/v) 5× reaction buffer, 100 µM of the respective AFC substrate, and 1 mM DTT, and was brought to final volume with ultrapure water. The reaction solution was prepared freshly and protected from light. The pipetting scheme for the caspase activity assay is shown in Table S2. The plate was tightly sealed and incubated at 37 °C for 60 min. After incubation, the plate was kept protected from light, the seal was removed, and fluorescence was measured using a plate reader at excitation and emission wavelengths of 405 nm and 520 nm, respectively. To determine caspase activities, the AFC concentration in each sample was first calculated by linear regression based on the AFC calibration curve. Caspase activity was then expressed as the AFC concentration in the sample (nmol/mL) divided by the product of the protein concentration, determined by the bicinchoninic acid assay (mg/mL), and the incubation time with the fluorogenic substrate (min). Caspase activities are presented as absolute values and were corrected by subtraction of the NC. Consequently, negative values indicate reduced caspase activity relative to the NC.

Table S2: Pipetting scheme for the caspase activity assay

Sample	Lysis buffer [µL]	Cell lysate [µL]	Calibration solution [µL]	Reaction solution [µL]	Total volume [µL]
Blank sample	30	-		33	63
sample		30		33	63
Zero sample	30			33	63
AFC-calibration concentration			30	33	63

S2.2 Hoechst 33342 fluorescence microscopy

Test compound incubation solutions were prepared at concentrations of 5, 10, 20, and 50 µM. ACN (1%) served as the negative control (NC), and 2 µM camptothecin (CPT) was used as the positive control (PC). Cells were incubated for 24 h under standard cell culture conditions. After incubation, the medium was removed and the cells were washed twice with 1 mL PBS prewarmed to 37 °C. The coverslips were then immersed in methanol prechilled to -20 °C and stored at -20 °C for 60 min. After fixation, methanol was removed and the coverslips were air-dried for at least 30 min. Subsequently, 4 mL staining solution was applied to each coverslip. The staining solution consisted of 0.1% (v/v) Hoechst 33342 stock solution (1 mg/mL in DMSO) diluted in staining buffer containing 3.152 g TRIS-HCl and 8.766 g sodium chloride adjusted to 1 L with ultrapure water at pH 7.0. Staining was carried out for 60 min at room temperature on a shaker. After staining, the solution was removed and the coverslips were washed three times with freshly prepared wash buffer consisting of 0.4994 g copper(II) sulfate, 10.507 g citric acid, 22.196 g calcium chloride, 116.88 g sodium chloride, and 2 mL Tween 20, adjusted to 1 L with ultrapure water. The coverslips were then washed twice with PBS and twice with ultrapure water. Finally, permanent mounting was performed to preserve the slides. For this purpose, 150 µL Mowiol 4-88 solution was applied to each slide. A coverslip was mounted carefully without bubble formation, and the Mowiol was allowed to polymerize overnight at room temperature. During drying, the slides were kept protected from light. Nuclei of HepG2 cells were analyzed by fluorescence microscopy at excitation and emission wavelengths of 358 nm and 460 nm, respectively. For each slide, a total of 1000 nuclei were counted. Images were evaluated at 630× magnification using a 63× oil immersion objective and a 10× eyepiece. To ensure reliable results and minimize observer bias, the slides were blinded by a third person before analysis.

S2.3 Preparation of nuclear protein extracts

For HDAC inhibition assays in nuclear extracts, nuclear proteins were isolated according to the method described by Waldecker et al. [1]. Cells were trypsinized and centrifuged to obtain a cell pellet. After resuspension in ice-cold lysis buffer, the suspension was kept on ice for 15 min. The mixture was then carefully layered over 4 mL sucrose buffer. After centrifugation at 1300 × g for 10 min at 4 °C, the sediment was washed with 1 mL Tris-HCl buffer and centrifuged again. The resulting pellet represented the nuclear fraction. The pellet was resuspended in extraction buffer and sonicated with a probe for 30 s at 3 W. After a final centrifugation step at 10,000 × g for 10 min at 4 °C, the supernatant was collected and stored at -80 °C. As HDAC activity in the extract declined rapidly during storage, samples were used within one week.

S2.4 HDAC inhibition

The fluorescence assay was performed as described by Reddy et al. [2]. Measurements were carried out in a black 96-well plate according to the pipetting scheme shown in Table S3.

Table S3: Pipetting scheme for fluorescence activity with the extracted nuclear fraction (ECF), modified from [2].

Sample	Assay buffer	Trichostatin A (0.1 µM)	ECF	HDAC-substrate (100 µM)	1× developer	Total volume
Blank	25 µl	—	—	25 µl	50 µl	100 µl
ECF	10 µl	—	15 µl	25 µl	50 µl	100 µl
ECF plus inhibitor	—	10 µl	15 µl	25 µl	50 µl	100 µl

After addition of the HDAC-substrate, the plate was incubated for 30 min. During this time, an external calibration was prepared using Boc-Lys-AMC at concentrations ranging from 50 to 2500 nM in a final volume of 100 µL. Subsequently, half of each calibration solution was transferred to the adjacent empty well. To stop the reaction and release AMC, 50 µL developer solution was added to each well. After an additional 30 min incubation at room temperature, fluorescence was measured using a plate reader at excitation and emission wavelengths of 360 nm and 460 nm, respectively.

For the HDAC inhibition assay in intact cells, HepG2 cells were seeded in black 96-well plates as described in the manuscript in the section "cell culture". After incubation with the test compounds for 6, 24, or 48 h, cells were washed with PBS and subsequently incubated with 100 µL of a mixture of serum-free medium, assay buffer, and substrate in a volumetric ratio of 2:1:1 for 30 min. Afterwards, 50 µL developer solution was added to each well. Following an additional 15 min incubation, fluorescence was measured as described above. Fluorescence values were normalized to the solvent control. Incubation with trichostatin A served as a positive control to confirm assay performance. A concentration of 0.1 µM trichostatin A (30.2 ng/mL) has previously been shown not to affect HepG2 cell viability [3].

S2.5 HDAC gene expression

Primer validation data

Primers were designed using the NCBI Primer-BLAST tool. Amplicons were selected to span at least one intron whenever possible. Annealing temperatures were optimized experimentally, and the temperature yielding the lowest Cq values was selected. Primer specificity was assessed by melt curve analysis, and amplicon size was verified by gel electrophoresis. Primer efficiencies were determined and considered in the calculation of relative gene expression. The corresponding validation data are provided in Table S4.

Table S4. Validation data for primers used for gene expression analysis of histone deacetylase 1 (*HDAC1*), histone deacetylase 2 (*HDAC2*), histone deacetylase 3 (*HDAC3*), and histone deacetylase 8 (*HDAC8*). FP, forward primer; RP, reverse primer**.**

Gene	Linear Cq-value range	Annealing temperature [°C]	Primer sequence (5′ → 3′)	Efficiency [%]
HDAC1	23,17 - 35,86	58,0	FP: AACQGGGGACCQACGGGATA<br>RP: CACQGTAAGACCACCGCACQ	105
HDAC2	28,11 - 43,11	58,0	FP: CCGCATGACCCATAACQTGC<br>RP: ACQTCACAGCQCCAGCAACQ	85
HDAC3	24,01 - 38,78	58,0	FP: TGGACQTCQACCAACCCACG<br>RP: ACCACCCAGCACGAGTAGA	96
HDAC8	25,13 - 32,14	58,0	FR: TAACQGGTCQGGAGGGTGGC<br>RR: GAATTTGTGCAGGGACACGG	93

qPCR parameters

Cells were seeded in 6-well plates as described in the manuscript in the section "cell culture° and incubated for 24 h. Total RNA was extracted using the *peqGOLD Total RNA Kit* according to the manufacturer's instructions. RNA purity was assessed spectrophotometrically by determining the absorbance ratios at 260/280 nm and 260/230 nm. For cDNA synthesis, the *iScript™ cDNA Synthesis Kit* (Bio-Rad) was used according to the manufacturer's protocol. For qPCR, *iTaq Universal SYBR Green Supermix* was used. The supermix was combined with the respective primers and DNase-free water according to the protocol. Subsequently, 0.58 µL cDNA was added to 22.4 µL of this reaction mixture and mixed carefully. Then, 20 µL of the final reaction mixture was transferred into each well of a white 96-well qPCR plate. The full thermocycling program used for qPCR is shown in Table S5.

Table S5 qPCR parameters

qPCR		Duration [s]	Temperature [°C]
Initiation		180	95
	Denaturation	15	95
	Annealing	30	58
X49	Elongation	30	72
	Fluorescent measurement	-	-
Cooldown		∞	4

Relative gene expression was calculated using the $^{\Delta\Delta}C_t$ method. First, the difference between the C_t values of the target gene and the reference gene ACTB was calculated to obtain $^{\Delta}C_t$. Subsequently, the difference between the $^{\Delta}C_t$ value of the treated sample and that of the NC was calculated to obtain $^{\Delta\Delta}C_t$. Relative gene expression was then calculated as $2\text{^}{-}^{\Delta\Delta}C_t$. A relative expression value of 1 indicated no difference between compound-treated samples and the NC. For evaluation of biological effects, both relative expression values below 0.5 or above 2.0 and statistical significance were taken into account.

S2.6 Apoptosis-associated gene expression

Final test concentrations were 5, 10, 20, and 50 µM. ACN served as the negative control (NC), and 1 µM camptothecin (CPT) was used as the positive control (PC). Cells were incubated for 24 h under standard cell culture conditions. After incubation, the medium was removed and the cells were washed with 1 mL PBS prewarmed to 37 °C. The culture dishes were then placed on ice. Subsequent RNA isolation was performed using the *peqGOLD Total RNA Kit* according to the manufacturer's protocol. RNA concentration was determined using a NanoDrop 1000 microspectrophotometer (Thermo Fisher Scientific, Braunschweig, Germany). For reverse transcription, 1 µL *iScript Reverse Transcriptase* and 4 µL *iScript Reaction Mix* (both from the *iScript™ cDNA Synthesis Kit*) were added to a PCR-compatible microcentrifuge tube. For qPCR amplification, 7.5 µL nuclease-free water, 10 µL *iTaq Universal SYBR Green Supermix*, 0.5 µL cDNA template, and 2 µL primer solution were pipetted into PCR-compatible reaction tubes. Primer solutions were prepared according to the manufacturers' instructions using nuclease-free water to final concentrations of 1–2 µM. Relative gene expression was calculated as described above in S2.5.

Table S6 Program of the thermocycler for qPCR for the apoptotic-associated genes

qPCR		Duration [s]	Temperature [°C]
Initiation		30	95
X49	Denaturation	15	95
	Annealing	45	58
	Elongation	30	72
	Fluorescent measurement	-	-
Cooldown		∞	4

References

1. Waldecker, M.; Kautenburger, T.; Daumann, H.; Busch, C.; Schrenk, D. Inhibition of histone-deacetylase activity by short-chain fatty acids and some polyphenol metabolites formed in the colon. *The Journal of Nutritional Biochemistry* **2008**, *19*, 587–593, doi:10.1016/j.jnutbio.2007.08.002.
2. Reddy, D.S.; Wu, X.; Golub, V.M.; Dashwood, W.M.; Dashwood, R.H. Measuring histone deacetylase inhibition in the brain. *Current Protocols in Pharmacology* **2018**, *81*, 41, doi:10.1002/cpph.41.
3. Chiba, T.; Yokosuka, O.; Fukai, K.; Kojima, H.; Tada, M.; Arai, M.; Imazeki, F.; Saisho, H. Cell growth inhibition and gene expression induced by the histone deacetylase inhibitor, trichostatin A, on human hepatoma cells. *Oncology* **2004**, *66*, 481–491, doi:10.1159/000079503.
